# Supplementary material for: Identification of photoperiod-induced specific miRNAs in the adrenal glands of Sunite sheep (Ovis aries)
Source: Front Vet Sci. 2022 Jul 22;9:888207. doi: 10.3389/fvets.2022.888207 (PMC9354845; doi:10.3389/fvets.2022.888207)
Supplement: Supplementary file 1 [file Data_Sheet_1.ZIP › Supplementary file/miRDeep2 file.zip!/miRDeep2 file/Know_Structure/SP42A3/oar-let-7i.pdf]

miRBase precursor : oar-let-7i
Total read count : 397383
oar-let-7i read count : 397207
remaining reads : 176

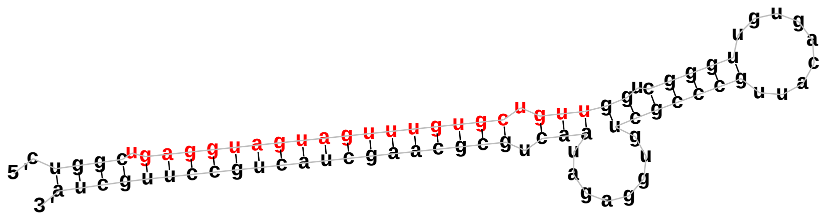

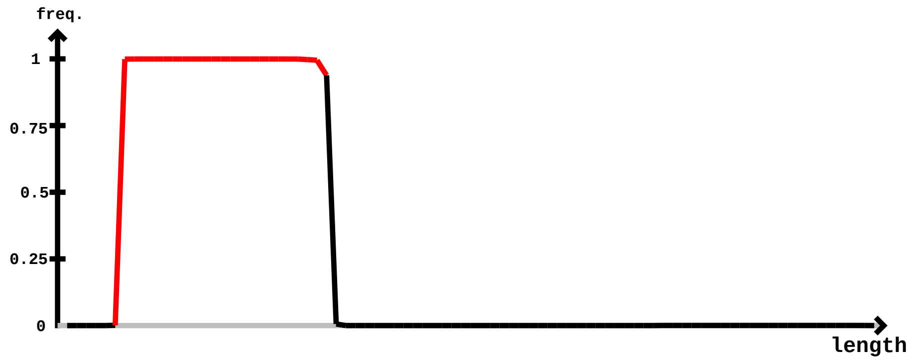


```
                oar-let-7i
5'- cuggcugagguaguaguuugugcuguuggucggguugugacauugcccgcuguggagauaacugcgcaagcuacugccuugcua   -3'    exp
    .((((.(((((((((((((((((.(((((.(((((.........)))))))........))).)))))))))))))))))))))   reads      mm         sample
    ....cugagguaguaguuugugcu............................................................   2          0          seq
    ....cugagguaguaguuugugcugu..........................................................   29         0          seq
    ....cugagguaguaguuugugcuguu.........................................................   376        0          seq
    ....cugagguaguaguuugugcuguug........................................................   3          0          seq
    .....ugagguaguaguuugug..............................................................   10         0          seq
    .....ugagguaguaguuugugA.............................................................   4          1          seq
    .....ugagguaguaguuugugc.............................................................   52         0          seq
    .....ugagguaguaguuugugG.............................................................   1          1          seq
    .....ugagguaguaguuugugcu............................................................   809        0          seq
    .....ugagguaguaguuugugcug...........................................................   963        0          seq
    .....ugagguaguaguuugugcugu..........................................................   22378      0          seq
    .....ugagguaguaguuugugcuguu.........................................................   370020     0          seq
    .....ugagguaguaguuugugcuguug........................................................   2384       0          seq
    .....ugagguaguaguuugugcuguugg.......................................................   8          0          seq
    ......gagguaguaguuugugcugu..........................................................   8          0          seq
    ......gagguaguaguuugugcuguu.........................................................   154        0          seq
    ......gagguaguaguuugugcuguug........................................................   3          0          seq
    .......agguaguaguuugugcuguu.........................................................   2          0          seq
    .........guaguaguuugugcugu..........................................................   1          0          seq
    .............................................................cugcgcaagcuacugccuug...   4          0          seq
    .............................................................cugcgcaagcuacugccuugc..   49         0          seq
    .............................................................cugcgcaagcuacugccuugcu.   119        0          seq
    .............................................................cugcgcaagcuacugccuugcua   3          0          seq
    ..............................................................ugcgcaagcuacugccuugcu.   1          0          seq
```
